# Insight into muscle physiology through understanding mechanisms of muscle pathology

**DOI:** 10.1007/s10974-015-9437-x

**Published:** 2015-12-15

**Authors:** Maria Jolanta Rędowicz, Joanna Moraczewska

**Affiliations:** Laboratory of Molecular Basis of Cell Motility, Department of Biochemistry, Nencki Institute of Experimental Biology, 3 Pasteur St., 02-093 Warsaw, Poland; Department of Biochemistry and Cell Biology, Faculty of Natural Sciences, Kazimierz Wielki University in Bydgoszcz, 12 Ks. J. Poniatowski St., 85-671 Bydgoszcz, Poland

This Special Issue of the Journal of Muscle Research and Cell Motility is devoted to the 44th European Muscle Conference (EMC) that took place in Warsaw, September 21–25, 2015. The EMC returned to Warsaw after almost four decades after the 7th muscle conference, which had been organized in 1978 by the late Professor Witold Drabikowski and his team at the Nencki Institute of Experimental Biology in Warsaw (Schaub 2010). The 44th EMC was organized by a group of Polish muscle scientists under the auspices of the Polish Biochemical Society. The main organizing institutions were the Nencki Institute of Experimental Biology, Faculty of Biology of the University of Warsaw, Department of Neurology of the Warsaw Medical University and Faculty of Natural Sciences of Kazimierz Wielki University in Bydgoszcz.

The main theme of the 44th EMC was Muscle Research in Health and Disease. It was the intention of the organizers to cover a wide range of topics focusing on muscle development and function, both in physiology and pathology. The program included the following sessions: “Molecular motors”, “Acto-myosin interactions”, “Muscle cytoskeleton”, “Muscle development and repair”, “Neuro-muscular interactions”, “Excitation–contraction coupling”, “Skeletal muscle diseases”, “Heart and heart failure”, “Smooth muscle in health and disease”, “Muscle metabolism and bioenergetics” and “Muscle exercise and plasticity”. The sessions were chaired by top scientists in these fields, including Polish muscle researchers. The organizers devoted two sessions to memorize outstanding muscle scientists who recently passed away. The session on “Acto-myosin interactions” was devoted to the memory of Professor Andrew Szent-György, and the session on “Smooth muscle in health and disease” was to the memory of Professor Renata Dąbrowska. The Young Scientist Session which preceded the Conference opening ceremony consisted of two parts: (i) How to stay motivated in science and (ii) Muscle biology: from genes to muscle. The opening lecture was presented by Professor Michael Rudnicki from Ottawa University, who pioneered studies elucidating the molecular basis of muscle development.

It was a great pleasure for us to host over 340 scientists from all over the world, nearly half of them were young researchers. There were 30 lectures delivered by the invited speakers who were the top researchers in their fields, 53 short oral talks presented mainly by young researchers and 169 poster presentations. The scientific quality of young researchers’ presentations were evaluated by the award committee, co-chaired by Dr. Christina Karatzaferi from University of Thessaly (Greece) and Dr. Stefan Galler from University of Salzburg (Austria). The committee granted 10 young researchers with a prize either for best poster (6 equal awards) or oral (4 equal awards) presentations. The awards were co-sponsored by the EMC organizers and the Journal of Muscle Research and Cell Motility published by Springer. Awards for best poster presentation went to: Sergej Pirkmajer from Slovenia, Danill Schchepkin from Russia, Przemysław Zakrzewski from Poland, Athanasios Moustogiannis from Greece, Jordan Blondelle from the USA and Josine M. de Winter from Netherlands. Awards for best talk went to Tahnee Kennedy from Australia, Zacharias Orfanos from Germany, Bahar Z. Camurdanoglu from Austria and Leonardo Nogara from Italy. Additionally, a special award granted by Dr. James R. Sellers went to Luca Fusi from United Kingdom for his talk at the “Molecular motors” session. The scientific program was accompanied by a social program that consisted of the Warsaw sight seeing tour, a Chopin concert and gala dinner. More details can be found at the Conference webpage—www.emc2015.org.pl.

Besides the EMC sessions, there were two satellite symposia on topics related to the Conference’s main theme: (i) Satellite Neuromuscular Symposium in honor of Gerta Vrbová organized by Professor Urzula Sławińska and (ii) EMC Satellite Meeting on the Muscle Synapse organized by Dr. Tomasz Prószyński. These events followed the Conference and were attended by numerous EMC participants.

We are grateful to the participants and the speakers who accepted our invitation to contribute with their work to create an excellent platform to discuss the latest achievements in muscle research and to initiate new collaborations. The responses from many Conference attendees who emphasized the high scientific level and excellent organization gave all members of the Organizing Committee a lot of satisfaction.

Speakers and chairmen of the EMC scientific sessions contributed to this Special Issue by submitting their papers. We are grateful to all of them that they put their efforts to write interesting articles and are sorry that others could not contribute, allowing us to keep our deadlines.

This issue consists of 7 reviews and 6 original papers, covering the topics of the Conference with an emphasis on translational studies, along with abstracts of the EMC short talks and poster presentations.

Three of the articles concern aspects related to muscle development and repair. Świerczek et al. (2015) summarize current knowledge on pluripotent stem cells (PSCs), comparing spontaneous and directed myogenic differentiation of PSCs as well as the protocols developed so far to facilitate this process. Kozakowska et al. (2015) discuss latest development on the role of oxidative stress and systems of enzymatic antioxidant defence in muscular regeneration after both acute injury and persistent muscular degeneration. Grabowska et al. (2015) in their experimental paper address the role of immune response during mice skeletal muscle regeneration showing the dependence of progress in muscle regeneration on the organism ability to properly react to inflammatory response.

Molecular mechanisms of skeletal muscle pathology are addressed in two articles. Malavaki et al. (2015) review the problem of disease-induced atrophy and disuse atrophy. The authors discuss the intricate network of interacting signalling pathways that seem to regulate disuse atrophy but also share common activation patterns with other types of muscle loss conditions, such as sarcopenia or cachexia. Kostera-Pruszczyk et al. (2015) provide more insight in BAG3-related fulminant myopathy and polyneuropathy, describing a patient presenting also symptoms of long QT syndrome.

Mechanisms of cardiomyopathy are addressed in two articles by the Szczesna-Cordary group as well as in the article by Burghardt et al. Huang and Szczesna-Cordary (2015) review the potential mechanisms of hypertrophic (HCM) or dilated (DCM) cardiomyopathy associated with mutations in the myosin regulatory (RLC) and essential (ELC) light chains. The authors also discuss the effects of mutations on myosin light chain phosphorylation and point to the fact that exogenous myosin light chain phosphorylation and/or pseudo-phosphorylation could serve as potential rescue tools to treat hypertrophy-related cardiac phenotypes. Gomes et al. (2015), performed an in-depth proteomic analysis of proteins expression in the hearts of transgenic mouse models of pathological (a mutation A57G in the myosin essential light chain; A57G hearts) and physiological (a deletion of 43 N-terminal amino acids in myosin regulatory light chain, Δ43 hearts) cardiac hypertrophy. The authors showed that 30 proteins were differentially expressed in Δ43 hearts versus A57G hearts, and the difference concerned mitochondrial proteins involved in metabolic processes, Ca^2+^-binding proteins, chaperones as well as proteins engaged in fatty acid metabolism. Burghardt et al. (2015) addressed the mechanisms of hypo- and hyper-contractility in cardiac and skeletal muscle that are associated with inheritable myopathies (IM), aging, and life-style. The authors concentrated on inheritable cardiomyopathies linked to the myosin motor. IM-linked mutations locate throughout myosin, impacting motor function. They used both high-throughput in vitro motility assays using super-resolution quantum dot particle tracking (Qdot assay) and in vivo single myosin detection in zebrafish embryos to analyse the deficiencies. The authors suggested that these approaches might enable linking phenotype to in vitro and in vivo myosin unitary mechanics by linking top-down and bottom-up modelling of human muscle disease.

The mechanisms of smooth muscle contraction are reviewed by Mills et al. (2015) who concentrated on the role for the Ca^2+^-dependent genistein-sensitive tyrosine kinase Pyk2 in tonic depolarization-induced contraction of vascular muscle.

The role of Ca^2+^ ions is also discussed by Csernoch and Jacquemond (2015), who provide an update on the established, the questioned and the unknown mechanisms regarding the role of phosphoinositides in skeletal muscle Ca^2+^ homeostasis and excitation–contraction (EC) coupling, particularly in differentiated skeletal muscle fibers. Another article addressing Ca^2+^-dependent EC coupling by Barone et al. (2015) reviews molecular interactions within the junctional SR (j-SR), consisting of the terminal cisternae that face the t-tubule at triads. The authors particularly discuss the role of junctophilins in structural organization of the proteins that are involved in excitation–contraction coupling and are located at the j-SR.

The problem of neuromuscular interactions has been addressed by Strack et al. (2015), who employed a recently developed longitudinal radioiodine assay to study turnover of acetylcholine receptors (AChR) at the endplate. The authors revealed profiles of AChR *de novo synthesis* and receptor incorporation into the postsynaptic membrane as well as a peculiar pattern of decay of pre-existing AChRs upon denervation. The observations seem to corroborate the existing model of a two-step AChRs stabilization.

Tropomyosin-dependent mechanisms of acto-myosin interactions, which are fundamental for muscle contraction, have been addressed by Rynkiewicz et al. (2015). The actin–tropomyosin interface is important for stabilization of the thin filament activation states. Using computational analyses the authors provide data which suggest that there is no need for tropomyosin to rotate around its axis in order to change its position on the actin filament, as it was proposed in earlier models (e.g. von der Ecken et al. 2015). The results allow the authors to suggest the mechanism for thin filament assembly, which may involve interplay between initially seeded tropomyosin molecules growing from distinct binding-sites on actin.

The analysis of the 44th EMC program as well as of the preliminary program of the next EMC, which will take place in Montpellier in 2016 and was presented in Warsaw by the French organizers, indicates that the focus of the muscle conferences shifts to include various clinical aspects of muscle function. This tendency has been continued for last decade and it was particularly visible at the meeting in Amsterdam in 2013. In our opinion, this does not mean that there is no more need to study the basics of muscle contraction in physiology, but rather that understanding mechanisms of pathology helps to get more insights into muscle physiology. Interestingly, new imaging techniques both in vitro and in vivo (for example Strack et al. and Burghardt et al.) and experimental models (for example zebrafish in Bughardt’s paper) are being exploited aiding in broadening our knowledge of the mechanisms of muscle contraction.
